# Development of Liposome-Based Hydrogel Patches Incorporating Essential Oils of African Plants and Deep Eutectic Solvents

**DOI:** 10.3390/gels11050364

**Published:** 2025-05-15

**Authors:** Wanhang Jiang, Sara Toufouki, Subhan Mahmood, Ali Ahmad, Alula Yohannes, Yang Xiang, Shun Yao

**Affiliations:** 1 School of Chemical Engineering, Sichuan University, Chengdu 610065, China; wanhang_jiang@126.com (W.J.); sara_toufouki@126.com (S.T.); engnr.chem.subhan@gmail.com (S.M.); aliahmad@stu.scu.edu.cn (A.A.); 2 College of Natural Science, Wolkite University, Wolkite City P.O. Box 07, Ethiopia; alulayhnns@gmail.com; 3 Children’s Drug Research Institute, Jianmin Group, Wuhan 430052, China

**Keywords:** hydrogel, nanoliposomes, essential oils, deep eutectic solvents, skin dressing, multifunctions

## Abstract

A nanoliposome-integrated polymeric hydrogel was developed for the controlled release of essential oils (Argania spinosa, Passiflora edulis). A deep eutectic solvent (DES) composed of betaine and phytic acid enhanced the solubility and stability of essential oils, facilitating uniform encapsulation within nanoliposomes. The hydrogel exhibited a swelling capacity of 100% and retained 51.7% of water after 7 h, ensuring prolonged hydration. Structural analysis confirmed a homogeneous dispersion of nanoliposomes, contributing to the gradual release of bioactive components. Additionally, the hydrogel demonstrated high mechanical strength (7.5 MPa), ensuring flexibility and durability. The polymeric network, formed by acrylamide, sodium alginate, and bentonite, provided a stable and elastic matrix, optimizing water retention and mechanical performance. The controlled diffusion mechanism of the nanoliposomes was validated through in vitro release studies, indicating Fickian-controlled release behavior. These findings highlight the potential of this polymeric hydrogel system as a functional material for skincare formulations, offering enhanced hydration and sustained bioactive delivery.

## 1. Introduction

As the largest human organ (surface area >2 m^2^ for adults), skin serves as the primary protective barrier. Its critical functions include maintaining homeostasis through thermoregulation, UV radiation protection, and defense against physical/chemical/microbial threats. Skin health represents a critical indicator of personal health [1]. As a prevalent dermatological manifestation, dry skin arises from compromised stratum corneum hydration homeostasis. Clinically characterized by desquamation and fine rhytides, this condition may predispose to premature skin aging through mechanisms involving immune barrier dysfunction and epidermal barrier integrity impairment. As the skin ages, it not only loses its biomechanical strength but also becomes increasingly prone to environmental damage [2], which in turn impairs its ability to heal wounds [2,3]. In addition, the skin also faces various problems such as dull color, dandruff, scars, wrinkles, bedsores, inflammation, etc., which constitute the needs of daily care for the body surface. Combined with growing consumer preferences for natural products, the continuous enhancement of hygiene standards within the cosmetics and health and wellness industries has prompted both domestic and international companies in the field of daily chemical products to intensify their research and development efforts on plant-derived ingredients [4]. Compared to those artificially synthesized components, active natural products are more friendly and safe and often have more comprehensive activities/effects to meet multiple nursing requirements.

In recent years, the use of plant-derived extracts in modern medicine and skincare has gained significant attention, while essential oils (EOs) are widely used in medicine, food, and cosmetics due to their antibacterial, antioxidant, anti-inflammatory, antiviral, moisturizing, and nourishing properties [5]. Among plant-derived oils, nut oils (e.g., argan oil) and fruit seed oils (e.g., passion fruit seed oil) have garnered increasing interest due to their richness in essential fatty acids, vitamins, and antioxidants. From North Africa, argan oil (Argania spinosa, AO) consists primarily of oleic and linoleic acids and is known for its exceptional moisturizing and reparative properties [6]. Meanwhile, passion fruit seed oil (Passiflora edulis, PFSO) from South Africa is rich in vitamins A and C [7], which help strengthen the skin barrier and provide soothing and antioxidant effects. However, the high volatility, instability, and penetration limitations of these essential oils hinder their broader application in skincare formulations [8]. To address these limitations, advanced nano-delivery systems, such as nano-liposome technology, have been explored to enhance their stability and bioavailability. Up to now, the EOs of rosemary, lemon eucalyptus, artemisia annua, and evening primrose multiflora [9] have been successfully encapsulated, whose compatibility and activities were significantly enhanced. These systems help reduce the exposure of active ingredients to environmental factors (e.g., water, oxygen, and light), minimize evaporation losses, and improve transdermal efficiency [10]. However, the strategy of encapsulating essential oils in nanoliposomes alone faces a series of challenges such as low water content, poor storage stability, organic solvent residue, and limited biocompatibility [11], etc. Besides that, the combinational use of EOs is rarely explored, which can enable them to complement each other’s strengths and weaknesses and play a synergistic role.

Hydrophilic gels, also known as hydrogels, are three-dimensional polymer networks capable of retaining large amounts of water [12], which have exhibited reversible physicochemical changes in response to external stimuli such as pH, temperature, ionic strength, enzymatic activity, and electric fields [12]. Depending on various applications, they can be formulated into various physical forms, including slabs, microparticles, nanoparticles, coatings, patches, and films. Compared to conventional formulations such as gels, creams, and emulsions for skincare, hydrogels exhibit superior capabilities in maintaining sustained therapeutic concentrations of active ingredients at targeted sites [13], enabling precise topical delivery with enhanced percutaneous penetration efficacy [12]. Hydrogels demonstrate superior moisture retention capabilities compared to cream while also allowing for unrestricted cellular migration. Additionally, their 3D polymeric structure offers enhanced tissue protection, in contrast to the lipid-based barrier properties characteristic of conventional creams [8]. Especially, a paper-free, sprayable skin mask was successfully developed, composed of a thermogel formed from nicotinamide-loaded biodegradable amphiphilic block copolymers [10]. Nevertheless, the solubility of essential oils in hydrogels is limited, which restricts the further development of their applications. As an effective green medium, deep eutectic solvents (DESs) may offer a promising solution to address this limitation [14].

As a class of low-melting-point mixtures composed of hydrogen bond donors and acceptors, DESs have recently garnered attention due to their designability, multifunction, and biocompatibility. In pharmaceutical applications, DESs exhibit exceptional drug solubilization capabilities, enhancing the solubility of poorly water-soluble active pharmaceutical ingredients (APIs) by 6–2200-fold compared to conventional solvents such as DMSO while also mitigating toxicity concerns [15]. Their tunable physicochemical properties, governed by the component ratios and hydrogen-bonding networks, enable the tailored synthesis of biodegradable polymers (e.g., poly(diol-co-citrate)) under mild conditions and the stabilization of thermolabile APIs [16]. DESs also advance functional nanomaterials by optimizing biocompatibility and drug-loading efficiency in graphene/DNA hybrids [17] and facilitating the green synthesis of antimicrobial metal–organic frameworks [18]. However, there are several aspects worthy of research and improvement, such as structural instability under aqueous conditions (where >50% water disrupts supramolecular organization) [19], as well as the effective use of those DESs with multifunctions (e.g., as active component, solvent, penetration enhancer, film-forming aid simultaneously, etc.) [20]. The existing challenges highlight the need for innovative delivery systems to harness the advantages of DESs. Our study introduces liposome-based hydrogel patches integrating DESs and African plant essential oils, using liposomal encapsulation to stabilize DESs against hydration-induced degradation, and synergizing with essential oils to enhance transdermal permeation.

As two famous representatives of African EOs, AO and PFSO were combined to be applied in this study for the first time, which were further solubilized with a DES composed of betaine and phytic acid and then encapsulated within nanoliposomes to enhance their stability and activities. The designed system aimed to facilitate their use and skin absorption and prolong their effectiveness, thereby providing new strategies and scientific foundations for the future development of skincare formulations. Guided by this strategy, the preparation of argan oil–passion fruit oil mixtures and their basic properties as well as key bioactivities were first investigated, and, then, the method was developed for the preparation of nanoliposomes encapsulating essential oils and DESs. After necessary characterizations, the obtained nanoliposomes were used in the preparation of hydrogel patches as designed. Finally, the comprehensive measurements and study on releasing behaviors of the hydrogel were performed. The incorporation of nanoliposomes was expected to effectively enhance the stability of the bioactive components within the hydrogels and improve their skin permeability and bioavailability. This whole work aimed to provide a useful strategy and scientific basis for advancing EO-involved skincare systems with multifunctions and good properties.

## 2. Results and Discussion

### 2.1. Spectral and Chromatographic Analysis of DES

**Figure 1:**
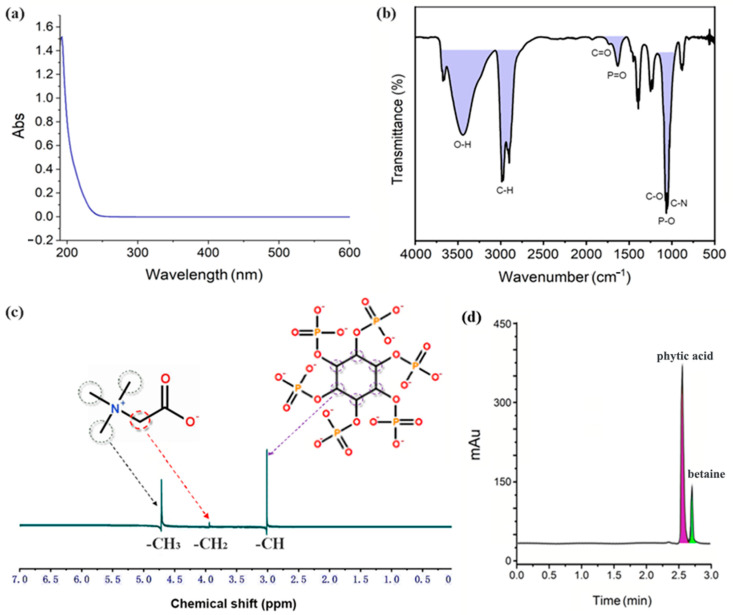
UV spectrum (**a**), FT-IR spectrum (**b**), 1H-NMR spectrum (**c**), and HPLC chromatogram (**d**) of the prepared DES.

The prepared betaine-phytic acid (1:3) is a yellow brown uniformly transparent DES [21], chosen for its optimal balance between enhancing skin permeability, stability, and controlled release. This DES also offers excellent biocompatibility, minimal skin irritation, and synergistic effects from the antioxidant properties of phytic acid and the skin-conditioning benefits of betaine, making it ideal for transdermal applications. Due to the lack of more effective UV-absorbing groups, it mainly produces strong absorbance around 200 nm (see Figure 1a), which provides a basis for the selection of detection wavelength and mobile phase in the following liquid chromatography quantification. Compared to the FT-IR spectra of its H-bond acceptor and donor as two components (phytic acid: the stretching vibration of O–H around 3350 cm^−1^, the stretching vibration of P=O at 1646 cm^−1^, the stretching vibration of P–O at 1125 cm^−1^, and the stretching vibration of C–O at 1143 cm^−1^; betaine: the stretching vibration of O–H around 3399 cm^−1^, the stretching vibration of C=O at 1615 cm^−1^, the bending vibration of C–H at 1478 cm^−1^, and the stretching vibration of C–N at 1053 cm^−1^; see Figures S1 and S2 in Supplemental Materials), Figure 1b indicates that the stretching vibration of P=O was shifted to 1636 cm^−1^ for the effect of intermolecular H-bonding, and the stretching vibration of C–N was shifted to 1058 cm^−1^ due to the electronegativity of anions together with the induction effect. In addition, the signals of phytic acid were obviously much stronger than those of betaine due to the high proportion of the former. The above signal attribution was based on previous studies [22,23].

For the ^1^H-NMR spectrum (400 MHz, D_2_O) of betaine-phytic acid (1:3) in Figure 1c, all the proton signals of the H-bond acceptor and donor, except the active hydrogen, appeared as expected, and no new signals were found. It proved that no chemical reactions occurred between them, and the DES formation only relied on their intermolecular interactions. In detail, the highest signal at 4.70 ppm belonged to –CH_3_ groups connected to nitrogen cation in betaine, the shortest peak at 3.93 ppm was contributed by –CH_2_ connected to nitrogen cation in betaine, and the medium peak at 3.03 ppm resulted from –CH groups in the ring of phytic acid. Due to not being key sites of intermolecular interactions, their chemical shift change was not significant.

Finally, the high-performance liquid chromatographic conditions for the prepared DES were explored. It was found that phytic acid and betaine were eluted successively instead of just one chromatographic peak appearing (see Figure 1d). It also proved that the formation of DES did not represent the synthesis of a new compound. Moreover, the flow rate had little effect on their peak resolution, so it was not taken as the object of investigation. The conditions were first attempted when the flow rate was 1.0 mL/min, the column temperature was 30 °C, and the mobile phase was a mixture of UP water and acetonitrile. The result indicated that, as the proportion of water in the mobile phase increased from 90%, the peaks corresponding to phytic acid and betaine were gradually separated. When the proportion of water reached 97%, the resolution of phytic acid and betaine was the best, and they could achieve baseline separation. When the mobile phase was changed to the mixture of UP water and methanol, the resolution became poorer compared to that resulting from the mobile phase of water–acetonitrile in the same proportion; the corresponding peaks of phytic acid and betaine partially overlapped. Besides that, methanol has strong end absorbance around 200 nm. Therefore, acetonitrile was finally chosen to replace methanol in the mobile phase. When the flow rate was set to 1.0 mL/min and the mobile phase was UP water–acetonitrile (97:3), the resolution of chromatographic peaks of phytic acid and betaine improved with increasing column temperature from 20 °C to 35 °C. The resolution improvement was not significant at higher temperatures, so the column temperature was set to 35 °C. As a result, the standard curves of betaine and phytic acid were y = 1.6621x + 0.0156 (R^2^ = 0.9990) and y = 1.1847x + 0.0015 (R^2^ = 0.9991), where y was the peak area, and x was the DES concentration (mg/mL).

### 2.2. Basic Properties and Activities of EO Mixtures

#### 2.2.1. Basic Properties

**Figure 2:**
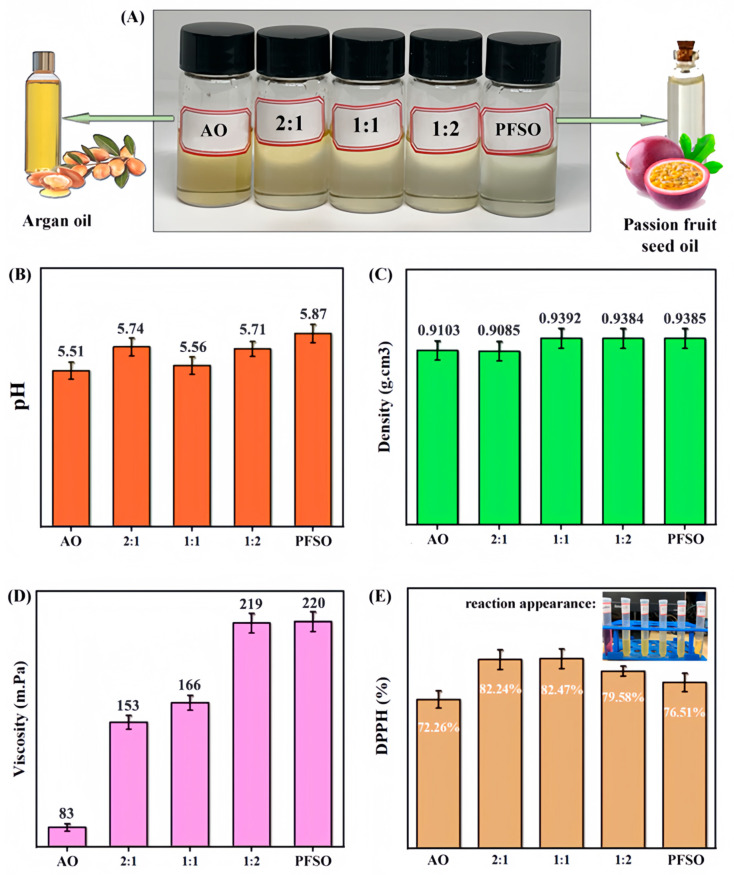
(**A**) Appearance of the AO-PFSO mixtures; (**B**) pH of the oil mixtures at different ratios; (**C**) densities of the oil mixtures at different ratios at 25 °C; (**D**) apparent viscosity values of the oil mixtures at different ratios at 25 °C; (**E**) percentage of free radical scavenging capacity of the oil mixtures. The inset shows the decolorization of the oil samples from purple to yellow in the DPPH assay, indicating the degree of antioxidant reduction.

It should be noted that the following research can not only provide basic data for the direct use of these essential oils but also is closely related to their preparation into nanoliposomes. As shown in Figure 2A, the appearance of all the samples containing blends of AO and PFSO in different proportions exhibited slight differences. Notably, the argan oil appeared more yellowish in color compared to the passion fruit seed oil. Moreover, the pH results in Figure 2B accord with the fact that most natural essential oils are acidic with a pH value between 5 and 8. The normal pH environment of the skin is usually between 4.5 and 5.5, which is a slightly acidic condition. The weakly acidic pH can help maintain the health of the skin. The preservation of a healthy stratum corneum (SC) and the integrity of the epidermal barrier are primarily dependent on the acidic pH of the skin surface. The homeostasis of the epidermal barrier, the cohesion, and integrity of the SC, the facilitation of proteolytic processes involved in desquamation, the metabolism of extracellular lipids, and the microbial colonization of the SC are all influenced by the acidity of the skin’s outermost layer. Both exogenous and endogenous factors contribute to variations in skin pH, which in turn affects the overall function of skin [24].

The density of natural essential oils is usually between 0.78 and 0.84 g/cm^3^, and there are also some special essential oils with densities higher than this range (especially those from fruits and kernels), such as cinnamon, cloves, rosehip, and sassafras oils, whose densities are close to or exceed 1 g/cm^3^ (density of water). As shown in Figure 2C, the density of the oil mixtures at various ratios is generally in the range of 0.9–1.0 g/cm^3^, which makes this type of essential oil prone to emulsification with water. At the same time, the density values of AO, PFSO, and their mixtures do not exhibit significant differences. The density of commercial argan oil was found to be consistent with the value reported by Taribak et al. [25]. Further analysis revealed that an increase in the concentration of passion fruit oil led to a rise in the overall density of the mixture.

In skincare products, viscosity is a critical factor that influences the texture, consistency, and overall sensory experience of the formulation. It affects the ease of application, absorption, and, ultimately, the efficacy of the product. An ideal gel formulation should strike a balance between sufficient fluidity to facilitate smooth application to the target area and adequate viscosity to ensure the maintenance of an effective concentration at the application site over time [25]. At around 20 °C, the apparent viscosity range of common plant EOs is roughly between 50 and 150 mPa·s, and some essential oils have lower viscosity (e.g., 35.4 mPa·s of coconut oil [26]). Here, the apparent viscosity of AO, PFSO, and their mixtures was evaluated, with the results presented in Figure 2D. The apparent viscosity values increased with higher concentrations of PFSO, which is the most viscous component among the samples tested. Specifically, PFSO exhibited an apparent viscosity of 220 mPa·s, significantly higher than that of argan oil (83 mPa·s). For the mixture at the 2:1 ratio, the apparent viscosity decreased to 153 mPa·s, while, for the 1:2 ratio, the apparent viscosity increased as the proportion of argan oil rose. These findings clearly demonstrate that AO has a lower apparent viscosity and that the overall apparent viscosity of the mixture depends on the relative proportions of the two EOs. Dissolving and diluting them with DES is beneficial for uniform dispersion and subsequent processing.

#### 2.2.2. Typical Bioactivities

The natural essential oils are well recognized for their antioxidant activity, which can help the skin resist the invasion of free radicals, delay skin aging, and promote skin repair and regeneration. Consequently, plant EOs are often regarded as a promising source for the development of novel bioactive components with potential applications in medicine, pharmaceuticals, cosmetics, and other industries [27]. In particular, argan oil is a rich source of essential polyunsaturated fatty acids, including high levels of oleic and linoleic acids. It is also notably abundant in tocopherols and polyphenols, both of which exhibit potent antioxidant properties [6]. Here, the antioxidant activity of mixtures of AO and PFEO is shown in Figure 2E, which was assessed using the stable free radical DPPH. Antioxidants, such as those found in these oils, play a crucial role in protecting the skin from damage and mitigating the skin aging process. It can be found that all the samples demonstrated the ability to reduce DPPH to yellow-colored diphenylpicrylhydrazine, with varying degrees of effectiveness. The extent of discoloration (from purple to yellow) correlates with antioxidant activity, with the strongest antioxidant activities (82.47% and 82.24%) observed in the ratios of 1:1 and 2:1, respectively, which were higher than those of the two essential oils alone. This reflects a certain degree of synergistic effect. For comparison, almond oil exhibited good antioxidant activity (e.g., 73.13%) as a component commonly used in skincare formulations [28], which was also noteworthy in relation to the EOs tested in this study.

**Figure 3:**
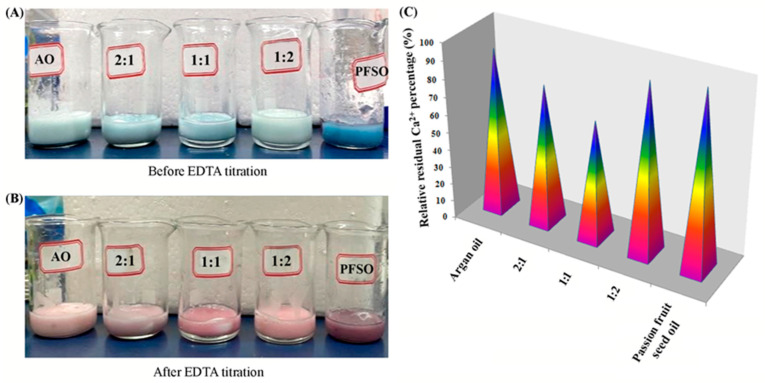
(**A**) Photographs of oil samples before and (**B**) after EDTA titration, from left to right: AO, 2:1 ratio, 1:1 ratio, 1:2 ratio, and PFSO. (**C**) Mass of calcium ions present in the oil samples. Note: the maximum level is treated as 100% to calculate the relative residual Ca^2+^ percentage.

Calcium is essential for regulating skin function, playing a significant role in the epidermal barrier [29,30]. Chemical exfoliation with strong calcium chelation activity can effectively rejuvenate skin cells and reduce enlarged conspicuous facial pores, which will make skin radiant and tender. Figure 3A,B show the actual status of the EO samples before and after titration. As indicated by the titration results in Figure 3C (details in Table S1 of Supplemental Materials), the mass of residual calcium present in each sample varies. Passion fruit oil exhibits the highest concentration of calcium ions, followed by the 1:2 mixed EOs, argan oil, the 2:1 mixed EOs, and, finally, the 1:1 mixed EOs, which have the lowest residual concentration of Ca^2+^. It indicated that the keratinization process of skin results could be blocked by virtue of the combination of 1:1 mixed EOs with calcium in pores; thus, the skin pores could be minimized temporarily. The increasing proportion of both AO and PFSO in the equivalent mixtures will lead to a higher concentration of calcium ions, weakening the performance of softening the skin.

Some studies have shown that EOs can be incorporated into skincare products to block UV radiation. In particular, oily substances are effective at forming a long-lasting sunscreen layer on the skin. Additionally, the moisturizing properties of oils help protect the skin from dryness caused by environmental factors such as wind and sun exposure. The sun protection factor (SPF) of the EOs samples was evaluated spectrophotometrically in vitro under UV-B light wavelengths (290–320 nm). Ultraviolet (UV) radiation, a component of the electromagnetic spectrum, is classified into three types: UV-A (320–400 nm), UV-B (290–320 nm), and UV-C (200–290 nm). UV-C radiation is effectively filtered by the stratospheric ozone layer and does not reach the Earth’s surface. In contrast, UV-A and UV-B rays penetrate the ozone layer and reach the surface, with the atmosphere filtering a greater proportion of UV-A radiation compared to UV-B. UV-B radiation, spanning from 290 to 320 nm, is one of the types of ultraviolet rays that can reach the Earth’s surface, although much of it is attenuated by the atmosphere. These rays play a significant role in the synthesis of melanin and the induction of sunburn. Therefore, protection from UV-B radiation is critical for preserving skin integrity and preventing damage [31,32,33].

Here, the ability of the oil samples to absorb UV radiation in the 280–320 nm range was assessed, and the sunscreen’s effectiveness was quantified using the sun protection factor (SPF). The values of EE(λ) × I(λ) [21] were summarized in Table S2 of Supplemental Materials, and Table 1 presents the SPF values for argan oil, passion fruit oil, and their mixtures, which were calculated using the same Mansur equation as Formula (3). The SPF values for the oil samples ranged from 1.43 to 1.68. The highest SPF value was observed for PFSO (1.68), followed by the AO-PFSO mixture at the 2:1 ratio (1.62), the 1:1 ratio (1.51), AO (1.50), and the 1:2 ratio (1.43), which are higher than that of eucalyptus (0.39), lavender (1.42), oregano (0.74), palmarosa (0.41), tea tree (1.04), and Japanese Yin Yang (0.59) EOs frequently used in skincare products [21]. These results indicate that PFSO demonstrated the strongest UV protection, with the EO mixtures showing a moderate sun protection effect. The difference between them is not so significant. Adding PFSO to AO is also beneficial in preventing skin color from darkening under light exposure when the latter is applied separately. Based on all the above results about the EO properties and activities, the AO-PFSO mixture at the 1:1 ratio was selected for the following preparation of nanoliposomes.

### 2.3. Characterization of Structure and Properties of Nanoliposomes

**Figure 4:**
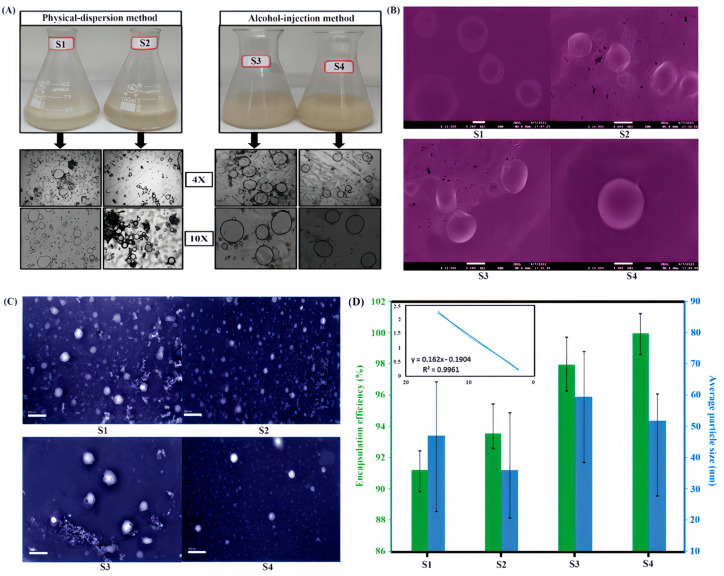
(**A**) Appearance of the prepared nanoliposomes and their optical microscope images in 40× and 100× magnification; (**B**) SEM and (**C**) TEM images of the prepared nanoliposomes as well as (**D**) average particle size and their encapsulation efficiency (inserted with concentration curve of Eos + DES).

The morphology of the prepared nanoliposomes (S1–S4) was first analyzed using optical microscopy, with representative images of the nanoliposomes and their microscopic structures presented in Figure 4A. The morphology of the nanoliposome formulations prepared via the physical dispersion method exhibited variations in both size and shape. Notably, not all the nanoliposomes displayed a spherical morphology; some were non-homogeneous and exhibited characteristics of multilamellar vesicles. In contrast, the S3 and S4 nanoliposomes exhibited relatively more uniform, unilamellar vesicles with a predominantly spherical shape and a more consistent size distribution compared to S1 and S2 nanoliposomes. These morphological differences can be attributed to the preparation method and the specific composition of the formulation. As a phospholipid with both hydrophilic and hydrophobic properties, lecithin plays a crucial role in the formation of these vesicles. Its amphiphilic nature allows lecithin to form stable lipid bilayers, which are essential for encapsulating hydrophobic compounds like essential oils. As the lecithin concentration in the formulation increases, the stability and encapsulation efficiency of the nanoliposomes improve, resulting in more uniform, unilamellar vesicles as observed in the S3 and S4 formulations. These findings contrast with those reported by other studies on nanoliposomes prepared using similar protocols. For instance, Khatibi et al. observed that nanoliposomes encapsulating *Zataria multiflora* Boiss. essential oil were larger and exhibited irregular shapes when they were prepared via the ethanol injection method [6]. Furthermore, the physical appearance of the formulations tended to acquire a more yellowish hue as the lecithin content in the formulation increased. Lecithin, which contains phospholipids, naturally imparts a yellow color, and, as its concentration in the mixtures rises, the intensity of the yellowish coloration becomes more pronounced.

Furthermore, Figure 4B and Figure 4C present SEM and TEM images of nanoliposomes (S1–S4) prepared using the two methods, respectively. Obviously, the majority of the particles exhibit a spherical shape, and various samples have various particle size distribution. S1 and S2 nanoliposomes have many small particles and even nonspherical particles. Additionally, the morphology of these nanoliposomes was further examined using TEM, revealing spherical forms with smooth surfaces as shown in the image of the sample of S4 nanoliposome. When comparing these findings with those previously published using the ethanol injection method, the results align well. For instance, current studies reported similar homogenous and spherical nanoliposomes [34,35,36], as the morphology of encapsulated particles, including size and shape, can be characterized using electron microscopy, with SEM detailing surface features and TEM revealing internal structures. The above results demonstrated that the preparation method employed for the S4 formulation was effective in producing high-quality nanoliposomes. By using ImageJ software (available at https://imagej.net/ij/download.html, accessed on 6 April 2025) to analyze the nanoparticle size distribution, the average particle size of the S4 sample was approximately obtained as 51.84 nm. Shanbnam [37] et al. reported that the use of propylene glycol led to the formation of nanoliposomes with the smallest mean particle size (92.03 nm) and a spherical morphology, making it the preferred co-surfactant. In comparison, the nanoliposomes synthesized in this study exhibited a significantly smaller particle size. Based on the comprehensive morphological evaluation, the nanoliposome based on S4 formulation was considered the most suitable for further studies and potential applications.

Encapsulation efficiency measurement is a crucial step in the characterization of liposomes [38]. To assess the incorporation rate of the active ingredients (EO mixture–DES system) into the lipid phase, UV spectroscopy was employed, which involved measuring the concentration of them in the supernatant following ultracentrifugation. A calibration curve at 280 nm was first established to evaluate the encapsulation efficiency according to the maximum absorbance wavelength of the EO mixture–DES system. To develop this curve, a series of working solutions of EOs and DES at varying concentrations were prepared using ethanol as the solvent. Figure 4D presents the calibration curve obtained and the encapsulation efficiency values for the nanoliposomes prepared using the physical dispersion and ethanol injection methods. The encapsulation efficiency of formulations S1–S4 was found to be showing a gradual upward trend, with values of 91.26%, 93.58%, 97.94%, and 99.95%, respectively. This suggests that variations in lecithin concentration have a certain impact on the encapsulation efficiency of the nanoliposomes (S2 > S1, S4 > S3). Consequently, it can be concluded that the lecithin content in the preparation of nanoliposomes is related to their encapsulation efficiency. When compared to the physical dispersion method, the nanoliposomes prepared via the ethanol injection method exhibited higher encapsulation efficiency, and the former also required a longer preparation time and involved more steps. Furthermore, all the tested formulations demonstrated exceptionally high encapsulation efficiencies above 90%. This is likely attributed to the hydrophobic nature of the encapsulated EOs, which exhibit a low affinity for the aqueous phase. This is further supported by the protective role of the hydrophobic phospholipid component in encapsulating and stabilizing the hydrophobic compounds. As a comparison, Isailović et al. [25] prepared liposomes encapsulating resveratrol using both the thin-film method and the pro-liposome method, achieving encapsulation efficiencies of 92.0% and 97.4%, respectively. Similarly, a study on the encapsulation of ibuprofen and flurbiprofen in nanoliposomes reported high encapsulation efficiencies of 54%, comparable to those observed in the present study [39]. On the basis of the above results, the S4 formulation was chosen for the preparation of the following hydrogel patches.

### 2.4. Characterizations and Measurements on Nanoliposome-Loaded Hydrogel Patches

#### 2.4.1. Appearance and Microstructure

**Figure 5:**
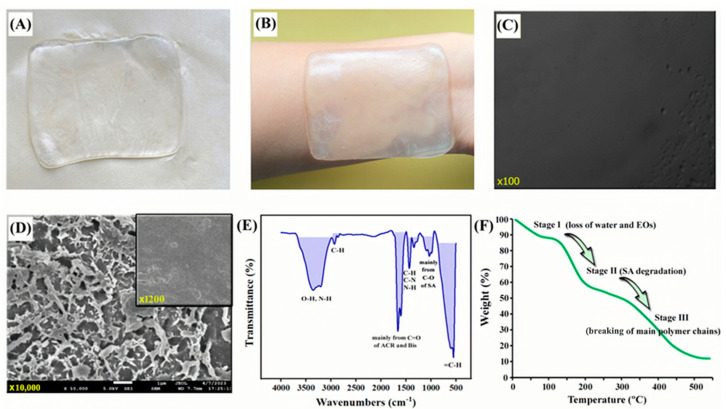
(**A**) The photo of the nanoliposome hydrogel patch; (**B**) the appearance of the prepared hydrogel attached on the human skin; (**C**) surface morphology in the optical microscope field of view (×100); (**D**) SEM images at different magnification (×1200 and ×10,000); (**E**) FT-IR ATR spectrum and (**F**) TGA results.

Visual inspection provides valuable insights into the quality and uniformity of hydrogel patches. The physical inspection of the prepared combination patches was conducted visually, with the results presented in Figure 5A,B. The hydrogel patches were colorless and transparent, with no visible air bubbles or cracks on the surface, which makes the users watch the skin status clearly. Furthermore, they exhibit a homogeneous composition. Upon direct application to the human skin, the patches adhere easily and do not leave any residue upon removal. Surface morphology in the optical microscope field of view (see Figure 5C) was also level and smooth, and there were no obvious cracks, grooves, or foreign matter. Based on these observations, the prepared hydrogel patches demonstrate satisfactory quality and are deemed suitable for further testing and characterization.

The microstructure of the hydrogel was further examined using SEM after the samples were dried in a vacuum. To enhance image clarity, the tested samples were coated with gold. As shown in Figure 5D, the originally smooth surface gradually reveals its interconnected network skeleton with an increase in the magnification. Similarly, Basu et al. synthesized an injectable hydrogel with sustained drug release by leveraging noncovalent interactions between DNA and nanosilicates. The incorporation of nanosilicates resulted in a more compact hydrogel network with reduced pore size, enhancing physical crosslinking and contributing to slower drug release [40]. Notably, the prepared patches contained numerous nanoliposomes encapsulating the essential oils (AO and PFSO). The size of the hydrogel patches was standardized to 1.5 cm × 1.5 cm, and the thickness of the hydrogel patches was measured at three different locations, yielding an average thickness of approximately 0.84 mm, which falls within the range of moderately thick hydrogel patches. This thickness was deemed suitable for various applications, offering an optimal balance between mechanical strength, durability, and active ingredient release.

#### 2.4.2. FT-IR and TGA

Here, FT-IR spectroscopy was also applied to identify functional groups present in the prepared hydrogel patches. As shown in Figure 5E, the peak at 3334 cm^−1^ corresponded to the stretching vibration of the hydroxyl and amino groups (–OH, –NH), indicating the presence of water, polysaccharides, and nitrogen-containing components in hydrogel patches. Moreover, the peak at 2918 cm^−1^ corresponded to asymmetric C-H stretching. The peak at 1658 cm^−1^ resulted from the C=O groups mainly in ACR and Bis, and the peaks in the range of 1450–1300 cm^−1^ were ascribed to C–N stretching vibration and the bending vibration of C–H and N–H. The broad peaks around 1020 cm^−1^ mainly belonged to the C–O stretching in SA. Finally, 550 cm^−1^ corresponded to the wavenumber of =C–H, respectively. These spectra were similar to previous reports. On the other hand, the weight losses of the hydrogel in different temperature regions are generally associated with a splitting of the main chain and thermal decomposition of the polymer [41,42]. When compared with the TGA results in the current study [43], the three weight loss stages of hydrogel patches were determined as shown in Figure 5F, which accorded with the main components in the whole system. The water and EOs were easily lost in the sample first, and, then, it was followed by the breaking of segments of mannuronic acid and glucuronic acid in SA in the temperature range of 100–225 °C. The degradation in the third stage (above 300 °C) accounts for the breaking of the main polymer chains.

#### 2.4.3. Swelling and Mechanical Properties

**Figure 6:**
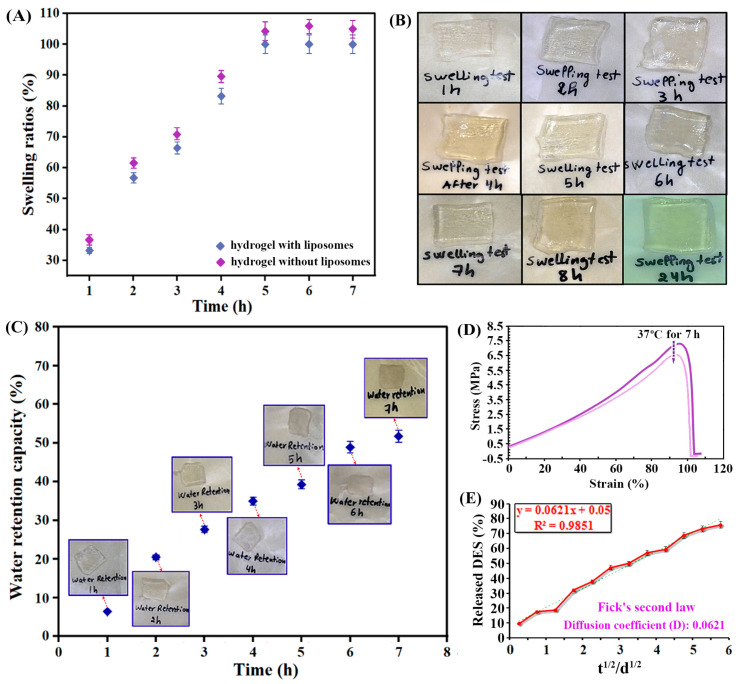
(**A**) Swelling ratio (the hydrogel sample without liposomes as control); (**B**) photographs of the prepared nanoliposome–hydrogel patch as a function of time (1–7 h) in PBS at 37 °C; (**C**) water retention capacity of the prepared nanoliposome–hydrogel patch as a function of time (1–7 h) at 37 °C; (**D**) stress–strain curves at room temperature and 37 °C; (**E**) DES release behavior in in vitro transdermal testing.

Fluid diffusion and swelling ratio variations are influenced by factors such as crosslink density, pore size, molecular weight, gel composition, and filler content. Swelling occurs as the polymer network occupies the available free volume within the sample until it reaches its maximum expansion, at which point equilibrium is achieved. Due to the flexibility of the polymer network, this expansion continues until osmotic pressures balance [44]. The swelling behavior of the nanoliposomal hydrogel patches developed in this study was investigated in a neutral medium (pH = 7.4) over a period of 1 to 7 h at 37 °C. Phosphate-buffered solution (PBS) was selected as the swelling medium, as it closely mimics the osmolality of physiological conditions [45]. The swelling ratio, which is defined as the percentage increase in volume relative to the dry hydrogel, reached its maximum value after 7 h, demonstrating the hydrogel capacity to absorb the swelling medium. In this study, the maximum swelling ratio observed was approximately 100%, meaning that the hydrogel’s volume increased by a factor of two after 7 h of exposure to the PBS. This behavior is consistent with the hydrogel’s intended application, as it ensures sufficient hydration without excessive swelling, which could compromise the stability and mechanical properties of the hydrogel. The results of the swelling capacity are presented in Figure 6A,B, showing the time-dependent swelling behavior and confirming the ability of the hydrogel to maintain its structural integrity over time. Besides that, it can be found from the comparison in Figure 6A that, because the liposome content in the whole system was limited, the swelling property did not show a great difference, so there was no more comparison on the basic properties between them.

In practical applications, it is essential to examine the water retention behavior of highly absorbent materials. In this study, the water retention capacity of the nanoliposomal hydrogel patch was assessed over time intervals ranging from 1 to 7 h. The results are shown in Figure 6C. After the first hour, the hydrogel lost 6.4% of its weight due to water absorption. By the second hour, the weight loss increased to 20.5%, and, after the third hour, it reached 27.7%. The hydrogel continued to lose water over time, with 35% loss after 4 h, 39.3% after 5 h, and 48.9% after 6 h. Interestingly, the hydrogel began to retain more water after the sixth hour, with only a slight increase in water loss between the sixth and seventh hours. After 7 h, the hydrogel had lost 51.7% of its weight in water. Gravimetric measurements are considered the gold standard for the measurement of water content [46]. This method involves determining the change in the weight of a sample before and after the removal of water. The difference in weight is attributed to the water content. Therefore, the weight loss measured during this test is attributed solely to water evaporation from the hydrogel matrix, thus directly reflecting its water retention capacity.

The prepared nanoliposomal hydrogel patches were found to be highly stretchable and flexible. When force was applied to break the hydrogel patch, it exhibited significant stretchability, and the tensiometer stretched it (length: 240 mm) under 5 N tension without breaking it. Measured with an electronic universal material tester, the stress–strain curves at room temperature and 37 °C are shown in Figure 6D. It can be found that the maximum stress was nearly 7.5 MPa, which would be reduced by 8% after remaining at 37 °C for 7 h. Similarly, Atefen et al. investigated the effect of curcumin liposome nanocarriers on the drug release behavior and mechanical properties of PVA/PEG hydrogels. Optimal nanoliposomes, prepared via thin-film hydration, were incorporated into the hydrogels, which were physically cross-linked through freeze–thaw cycles. Their study reported a maximum tensile stress below 1 MPa [47]. Furthermore, upon repeatedly folding the patch at the same spot on the skin more than 100 times, no cracks or defects were observed. These observations underscore the remarkable flexibility and durability of the hydrogel patches.

#### 2.4.4. Skin Adaptability and DES Release

When formulating skincare products, it is crucial to consider their pH effect on the skin. Regulating skin pH is an essential and controllable factor that can enhance skin penetration while also ensuring the stability, efficacy, and safety of products for direct skin application [34]. The skin serves as the first line of defense against external aggressions, and its microbiome and structural integrity are delicately maintained by various factors. Alterations in skin pH can lead to pathogenic effects, including compromised skin barrier function and increased bacterial colonization [34]. By using an intelligent skin tester, the pH of the hydrogel patch was measured at 5.57 at room temperature. Given that the normal pH range for skin is typically between 4.5 and 5.5, the measured pH of 5.57 is considered to fall within an acceptable range. Also, by using an intelligent skin tester, the water content of the skin increased from 48% to 54% after contact for half an hour, and oil content of the skin increased from 27% to 35%, indicating that the hydrogel patch exerts a hydrating effect on the skin. No significant changes were observed in the skin color and appearance, suggesting that the patch did not induce irritation and is likely safe for use. Due to the fact that the relevant components are commonly used in transdermal systems and no additional substances with obvious irritation or sensitization were used, no significant skin adverse reactions were found within 12 h (this patch was designed to be changed daily and used during sleep). These findings support the safety and efficacy of the hydrogel patch in promoting skin hydration without causing any adverse effects. Finally, removability can also reflect skin adaptability of the patches; if the adhesion is too strong, it may cause skin surface damage during the removal process. After the comparison with a commercial hydrogel patch through the investigation in Section 4.8.4, the difference in adhesion was not great (commercial hydrogel patch: 18.3 ± 0.5 g; our hydrogel patch: 15.8 ± 0.3 g). If it aimed to make peeling easier, a small volume of water was suggested to be dropped on it for its swelling property.

As introduced in Section 4.8.7, the two kinds of African essential oils have complex chemical components, so it is not easy to choose a well-recognized single compound for quantitative analysis in the study of in vitro releasing behaviors. Therefore, the DES of betaine–phytic acid (1:3) was chosen as the marker of a released object for investigation to demonstrate that the relevant components can be released, which was proven for its bioactivities and biocompatibility for skin in a previous study [21]. Plotting the percentage of DES release against the square root of release time, the release curve of DES is shown in Figure 6E. It can be seen that there is an approximate linear relationship between the two parameters, which conforms to Fick’s second law. Therefore, the release of the DES from hydrogel patches is mainly controlled by the diffusion mechanism, which is in accordance with the releasing behaviors of perillyl alcohol from its related hydrogel and emulsion [48]. Moreover, the permeability coefficient was determined based on the Franz diffusion cell as 1.12 × 10^−3^ cm/s. In the above mechanism, active molecules diffused through the polymer barrier in a concentration gradient, were then desorbed from the polymer, and diffused into body fluids or media.

## 3. Conclusions

This work presents a groundbreaking nano-liposome hydrogel patch engineered for advanced skincare applications, integrating argan oil, passion fruit oil, and a phytic acid–betaine deep eutectic solvent (DES). In our study, the hydrogel patches are designed to be “ready-to-use” immediately after production, following a vacuum drying step. This drying process ensures that the patches maintain their physicochemical integrity and sterility, making them suitable for further application in skincare. The hydrogel demonstrated exceptional hydration properties, achieving a swelling ratio of 100% and sustaining 51.7% moisture retention over 7 h, highlighting its efficacy in prolonged skin protection. The 100% swelling ratio indicates that the hydrogel can efficiently absorb and retain moisture, ensuring optimal hydration without excessive expansion that could compromise its mechanical integrity. This behavior is crucial for skincare applications, where long-lasting hydration is necessary for improving skin texture and appearance. Formulated with biocompatible polymers, specifically acrylamide and sodium alginate, combined with bentonite for structural reinforcement, the material, as an advanced platform designed for skincare, demonstrated robust mechanical properties, evidenced by a tensile strength of 7.5 MPa, as well as excellent flexibility. These characteristics ensure the system’s durability and comfort during prolonged direct contact with body skin. Notably, the encapsulation of bioactive oils within nanoliposomes significantly improved colloidal stability, transdermal permeability, and enabled sustained release kinetics. Collectively, these features not only promote the efficient and targeted transdermal delivery of hydrophobic actives but also markedly reduce issues such as burst release or rapid degradation. Therefore, the synergistic use of DESs and natural products within a polymer matrix holds strong potential for versatile applications in the controlled delivery of essential oils, skincare, and wearable bioactive interfaces, offering enhanced bioavailability, minimized biological disruption, lower skin irritation potential, and improved human compliance through its tailored release profiles and superior material performance.

## 4. Materials and Methods

### 4.1. Reagents and Materials

A variety of chemicals were procured from Kelong Chemicals Co., Ltd. (Chengdu, China), including ethanol (99.7%), 2,2-diphenyl-1-picrylhydrazyl (DPPH, 96%), ethylenediaminetetraacetic acid (EDTA, AR), sodium hydroxide (NaOH, AR), trichloromethane (99%), methanol (99%), sodium chloride (99.5%), calcium chloride (96%), N,N,N′,N′-Tetramethyl (96%), acrylamide (99%) and ethylenediamine. Furthermore, lecithin (soybean) (R-D) was obtained from Macklin Biochemical Co., Ltd. (Shanghai, China), while cholesterol (99%) was sourced from Rhawn Reagent Co., Ltd. (Shanghai, China). Betaine (98%), bentonite (R-D), sodium alginate (R-D), ammonium peroxydisulfate (98.5%), N,N-methylene-bis-acrylamide (99%), and phytic acid (97%) were provided by Aladdin Biochemical Technology Co., Ltd. (Shanghai, China), which also provided Eriochrome Black T (96%). Among above reagents, sodium alginate, bentonite, and acrylamide were basic ingredients for the hydrogel formulation. All the chemical reagents were employed in their original form without any further modifications. Ultrapure (UP) water was used as the experimental water and prepared by using a water purification system from Millipore Co., Ltd. (Bedford, MA, USA). Argan oil (AO) was sourced from Rahiq lhayat in Morocco, and passion fruit seed oil (PFSO) of South Africa was obtained from El Aroma in Germany (both > 90%), respectively.

### 4.2. Instruments

The main experimental instruments used in this study include a TU-1810 ultraviolet (UV) spectrometer from Purkinje General Instrument Co., Ltd. (Beijing, China) and a JSM-7001F scanning electron microscope (SEM) from JEOL Co., Ltd. (Tokyo, Japan). Additional instruments include the N-117M optical microscope (×40–100, Novel Optics, Ningbo, China), the H-600 negative-stain transmission electron microscope (TEM, Hitachi, Tokyo, Japan), and a KS-400KDE ultrasonicator (Jielimei Ultrasonic Inc., Kunshan, China). The proton nuclear magnetic resonance (1H NMR) spectrum of the diluted samples with deuterated solvent was recorded at room temperature using a JNM-ECZ400s/L1 spectrometer (400 MHz, JEOL, Tokyo, Japan). A Spectrum Two L1600300 Spectrometer (Perkin Elmer, Waltham, MA, USA) was applied to record FT-IR in the range of 4000–450 cm^−1^. Finally, a ZP-5 HNA tensile testing machine (±0.001 N; Fuma Electrical Instruments Inc., Dongguan, China), 5967 electronic universal material tester (Instron Corporation, Boston, MA, USA), and HTG-2 microcomputer differential thermal balance (Hengjiu Scientific Instrument Factory, Beijing, China) were employed for hydrogel characterization. Skin detection was completed with RBX-916 type intelligent skin tester (Real Bubee Medical Instrument Inc., Ningbo, China).

### 4.3. Preparation, Identification and Quantitation of DES

The DES was prepared by heating the betaine and phytic acid in specific molar ratio (betaine–phytic acid = 1:3) at 60 °C in water bath (±0.1 °C) until a clear liquid was formed [21]. Then, the mixture was taken out from the bath, and it was kept under vacuum in 50 °C for 24 h before use, which was further confirmed by UV, IR, and nuclear magnetic resonance (NMR) spectroscopy. In order to complete its purity and quantitative analysis, the UltiMate3000 high-performance liquid chromatography (DIONEX Co., Sunnyvale, CA, USA) was used according to the following conditions: 4.6 × 250 mm C_18_ column (5 μm, Welch Materials, West Haven, CT, USA) as stationary phase, the mixture of acetonitrile and water (3:97, *V*/*V*) as mobile phase 35 °C as column temperature, 1 mL/min as flow rate, 20 μL as injected sample volume, and 200 nm as the detection wavelength according to the UV full wavelength scanning.

### 4.4. Preparation of Argan Oil-Passion Fruit Oil Mixtures and Their Basic Properties

AO and PFSO were prepared in different volume ratios (2:1, 1:1, 1:2) using simple mixing method followed by stirring (300 rpm) for 30 min to ensure the thorough dispersion of the two EOs. The oil mixtures were kept at room temperature for the following characterization on pH, density, and apparent viscosity.

#### 4.4.1. Determination on pH of the Oil Mixtures

The chemical activity of hydrogen ions in solution was first measured by pH (hydrogen potential). The pH determines whether a sample is basic or acidic, and it is closely associated with its potential influence on skin. After inserting the pH-meter electrode directly into the EO mixtures, the ionic acidity of the product to be analyzed was characterized after the data were stable. This experiment was repeated three times to obtain the results.

#### 4.4.2. Determination on Density of the Oil Mixtures

The density of oil mixtures was determined by the ratio between the 10 mL of oil mixtures and the mass of the same volume of UP water taken at the same temperature. This experiment was repeated three times to obtain the results.

The density of each sample was calculated by using Formula (1) [49]:

(1) $$\rho_{M}=\rho_{w}\frac{\left(m_{M}-m_{p}\right)}{\left(m_{w}-m_{p}\right)}$$

where $\rho_{M}$ is density of the oil mixtures, g/cm^3^; $\rho_{w}$ is the density of water at 25 °C, which is 0.9970 g/cm^3^; m_p_ is the weight of the empty pycnometer, g; m_w_ is the weight of pycnometer filled with water, and m_M_ is the weight of pycnometer filled with the oil mixtures, g.

#### 4.4.3. Determination on Apparent Viscosity of the Oil Mixtures

All the measurements were performed using a STM-IV type viscometer (Meiyu Instrumental Inc., Shanghai, China) at 25 °C by inserting the spindle of viscometer directly into the sample. The mark on the mixing blade shaft was level with the liquid surface, and its rotational speed was 200 rpm. The measurements were performed in triplicate, and the average values were calculated. This experiment was repeated three times to obtain the results.

### 4.5. Key Bioactivities of Argan Oil–Passion Fruit Seed Oil Mixtures

#### 4.5.1. Diphenyl-1-Picrylhydrazyl Antioxidant Activity

The antioxidant activity (AA%) was determined using 2,2′-diphenyl-1-picrylhydrazyl (DPPH) method [50]. Briefly, 200 μL of different ratios of the essential oil mixtures was mixed with 2.8 mL of ethanol and then mixed thoroughly; after that, 2 mL of 0.004% ethanoic DPPH• solution was added. The mixtures were well shaken and kept at room temperature in the dark for 30 min. The absorbance of each mixture was measured at 517 nm using UV–Vis spectrophotometer. Ethanol was used as a negative control, and the radical scavenging activity (RSA) was calculated as a percentage of DPPH• discoloration by using Formula (2).

(2) $$AA\%=\frac{\left(A_{c}-A_{s}\right)}{A_{c}}\times 100$$

where A_c_ is the control reaction absorbance that contains all the tested reagents except the essential oils; A_s_ is the absorbance of tested samples. This experiment was repeated three times to obtain the results.

#### 4.5.2. Ca^2+^-Binding Activity (Antikeratotic Activity)

Based on current report [51], complexometric volumetric titrations with the ethylenediamine tetra-acetic acid (EDTA) were used to measure the quantity of calcium in oil samples. For 5 mL sample of the oil, a 5 mol/L NaOH solution was added until the mixture became strongly alkaline (around pH 12). Besides that, 2 drops of 1% (*W*/*V*) Eriochrome Black T indicator solution were further added into each sample, and they were titrated with 0.01 mol/LEDTA. When the system color was changed from pink to blue, the titration was complete. The residual concentration of calcium was determined after the titration to evaluate the Ca^2+^-binding activity of each sample. This experiment was repeated three times to obtain the results.

#### 4.5.3. Sun Factor Protection Activity

The measurement of the sun factor protection (SFP) activity was used to assess the photoprotective impact of oil mixtures with different compositions, and the samples were prepared by weighing 0.1 g of the tested oil mixture. The precisely weighed oils were mixed with 10 mL of ethanol under the radiation of 100 W ultrasound waves until homogenization, and the homogenous mixture was tested three times by using the spectrophotometer at 5 nm intervals within the wavelength range of 290–320 nm. The obtained values were calculated using the Mansur equation as Formula (3) [14,15,16,17].

(3) $$SPF=CF\sum_{290}^{320}EE(\lambda )I\left(\lambda \right)Abs\left(\lambda \right)$$

where EE(λ) is the erythemal effect spectrum, I(λ) is the solar intensity spectrum, and Abs is the absorbance of essential oils and their mixtures. CF is the correction factor (=10). The value of EE(λ) × I(λ) is constant determined by Sayre et al. [18]. This experiment was repeated three times to obtain the results.

### 4.6. Preparation and Characterization of Nanoliposomes Encapsulating Two EOs and DES

#### 4.6.1. Preparation of Essential Oils and DES-Encapsulated Nanoliposomes

**Figure 7:**
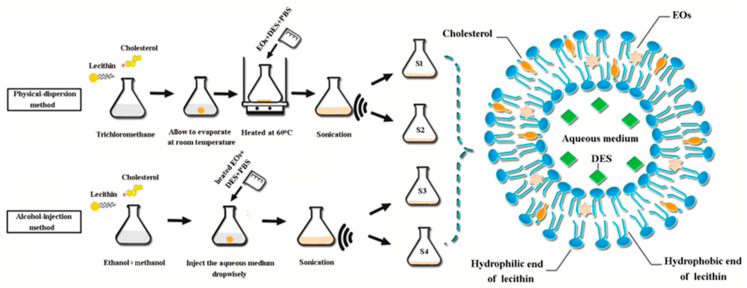
Diagram of preparation process for EO-encapsulated nanoliposomes in two ways.

In this section, the nanoliposomes were prepared by physical solid dispersion and ethanol injection methods to encapsulate the mixture of two essential oils, which are two commonly used methods in current studies. By comparing the appearance, encapsulation performance, energy consumption, and operational time required for each method, it was aimed to explore the most efficient way to prepare hydrogel patch nanoliposomes. For simple comparison, Figure 7 includes the main procedures of two methods; at the same time, Table 2 provides the details of ingredients for the two preparation methods.

(1) Physical-dispersion method

Nanoliposomes were prepared by solid dispersion method described previously by Shivhare [52]; different quantities of lecithin were used, while the cholesterol quantity was kept constant. The lipids were dissolved in 5 mL of trichloromethane and then transferred to a flat-bottom conical flask, then, they were evaporated at room temperature for 1 day without disturbing them to form a lipid layer. The oil mixture and DES were mixed with 50 mL of PBS buffer (pH 7.4) and ultrasonicated (500 W) to form a uniform emulsion, which acted as an aqueous medium. The flask containing the lipid film was tilted to one side, and the aqueous medium was introduced down the side of the flask and slowly returned to its upright position. The flask was then placed on the water bath and maintained at 60 °C for 3 h to ensure complete hydration of the lipid film. The formulations were subjected to ultrasonication (500 W) to reduce the size of the formed nanoliposomes. Finally, the formed nanoliposome suspensions were marked as S1 and S2 and were stored at 4 °C.

(2) Alcohol-injection method

Differently, S3 and S4 were prepared by alcohol-injection method [53], and the lipids were dissolved in ethanol and methanol. The aqueous medium was made by mixing oil mixture and the DES in 50 mL of PBS as performed previously, which was heated to 60 °C in the water bath. The heated aqueous medium was then introduced dropwisely into the lipid solution, triggering the spontaneous formation of nanoliposomes through interfacial self-assembly at the aqueous-lipid phase boundary. The nanoliposome suspensions were subjected to ultrasonication (500 W) to reduce the size of the formed nanoliposomes and stored at 4 °C.

#### 4.6.2. Characterization of Essential Oils and DES-Encapsulated Nanoliposomes

The following studies were based on current reports [54,55]. Firstly, a slide was prepared by placing a droplet of the prepared nanoliposome suspension on a glass slide, and cover slip was placed over it. And this slide was viewed under optical microscope at 40× and 100× magnification. Photographs were taken to the prepared slides using digital camera. Then, the structure of the diluted nanoliposome suspension was observed by Negative-stain transmission electron microscope (TEM). Briefly, a droplet of nanoliposome suspension was placed onto copper grids, and the excess was removed using filter paper after 3 min. The TEM was then used to evaluate negatively stained nanoliposomal sample at different magnification. Moreover, the SEM was further used to observe the appearance and morphology of nanoliposomes at high magnitude. The diluted samples were made by mixing 100 μL of nanoliposome suspension with 900 μL of PBS (pH 7.4), and, then, placing one droplet of the produced samples on a clean glass sheet, the sample was then placed on aluminum stubs and finally coated with gold. Finally, the efficacy of encapsulation was determined using UV spectroscopy. Briefly, after the maturation of nanoliposomes, the mixture was further centrifuged (15,000 rpm, 20 min). The supernatant was separated, and its absorbance was measured at 280 nm as maximum absorbance wavelength (see Figure S3). The encapsulation efficacy (EE, %) was calculated as Formula (4).

(4) $$EE\;(\%)=\frac{Total\left({EO}_{1}+{DES}_{1}\right)–\left({EO}_{2}+{DES}_{2}\right)}{Total\left({EO}_{1}+{DES}_{1}\right)}\times 100,$$

where EO_1_ refers to the concentration of argan oil and passion fruit oil used to prepare the nanoliposomes; DES_1_ refers to the concentration of DES used to prepare the nanoliposomes; EO_2_ refers to the concentration of argan oil and passion fruit oil in the supernatant; and DES_2_ refers to the concentration of DES in the supernatant. This experiment was repeated three times to obtain the results.

### 4.7. Preparation of Hydrogel Patches Containing the Formed Nanoliposomes

The hydrogel was prepared by mixing sodium alginate (SA) with UP water (1:50, *W*/*W*) under magnetic stirring for 30 min to ensure complete dissolution. Then, bentonite was dispersed in ultrapure water at a mass ratio of 1:100 and subsequently incorporated into the sodium alginate solution. The mixture was stirred under magnetic agitation for another 30 min in a 40 °C water bath (±0.1 °C). Next, 2 mL of the previously prepared EOs-DES nanoliposome was added into the mixture of sodium alginate and bentonite. Following this, 15 g of acrylamide (ACR) was added and mixed thoroughly, and 0.01 g of N,N′-methylene-bis-acrylamide (Bis) in 10 mL of UP wat was added to the solution. After thorough mixing, ammonium persulfate (APS, 10 mg/mL) and N,N,N′,N′-tetramethylethylenediamine (TEMED, 0.76 mg/mL) were added at a mass ratio of 1:2 to initiate crosslinking and gelation. The reaction proceeded at 60.0 ± 0.1 °C for 0.5–1 h to ensure uniform crosslinking and gelation. The viscous product was immediately transferred into the clean mold, cooled to room temperature for structural stabilization and finally demolded. Post-treatment procedures included vacuum drying (50 °C, 3 h), UV sterilization (60 min, 254 nm), sectioning into standardized specimens, and vacuum-sealed storage to preserve physicochemical integrity and sterility, successively. Post-treatment, the hydrogel patches were ready for immediate use in actual applications. For specific experiments in the following contents (such as swelling tests), the hydrogel patches needed to be immersed in phosphate-buffered saline (PBS) for up to 24 h to observe their swelling behavior and achieve the desired hydration state.

### 4.8. Characterization and Testing on the Prepared Hydrogel Patches

The prepared hydrogel patches were first evaluated through visual inspection for color, clarity, flexibility, and smoothness. Then, the following measurements and testing were performed for their main characteristics and properties. Each of the following experiments was repeated three times to obtain the results.

#### 4.8.1. Thickness Measurement

The thickness of hydrogel patches was determined using digital caliper (±0.01 cm) at three different places of each patch; the patch was placed between the two jaws of the calipers, which were gently closed until the patch was lightly compressed. Then, the thickness values on the digital display were recorded, and the mean value (n = 3) was calculated as final data.

#### 4.8.2. SEM Observation

The morphology of the hydrogel patches containing nanoliposomes was examined using SEM. Prior to observation, the hydrogel patches were vacuum-dried for 10 min, cut to reveal the internal structure, and then coated with gold by sputtering after drying. SEM images were observed at magnifications of 1200× and 6000×, respectively. It is essential for analyzing the detailed microstructure of materials at high resolution, enabling the visualization of nanoliposome distribution, size, and interaction with the hydrogel matrix.

#### 4.8.3. FT-IR Spectral and Thermogravimetric Analysis (TGA)

FT-IR spectrophotometer analysis was performed on the prepared dried hydrogel patches. The attenuated total reflection (ATR) mode of operation was used with the spectrophotometer, which can directly analyze solid samples without preparing transparent disks, simplify the sample processing process, and directly reveal the microstructure of the sample surface. The analysis was carried out in the wavenumber range from 4000 cm^−1^ to 500 cm^−1^. In order to confirm the basic composition of the hydrogel patches, TGA was carried out, and the sample was heated from 35 °C under an atmosphere of air at a constant temperature rate of 10 °C/h.

#### 4.8.4. Tensile, Folding Strength, and Removability Analysis

To determine the tensile strength of hydrogel patches, they were mounted in the tensile testing machine and subjected to a controlled stress until the hydrogel patch broke. The folding endurance of the patches was tested by manually folding one patch repeatedly at the same spot until it either broke or developed visible cracks, which was assessed to indicate good patch quality [53]. This is crucial to determine whether the sample can withstand folding during applications. The value of folding endurance was determined by how many times the patches could be folded in the same location without breaking. Finally, the universal material tester was applied to obtain the tensile stress–strain curves at room temperature and 37 °C. Finally, the adhesion of patches with the size of 1.2 × 1.2 cm^2^ was measured according to General Principles in the fourth part of Chinese Pharmacopoeia (2020 edition), which reflected their removability, and the commercial E-106 type hydrogel patch (Haiyang Biotechnology Co., Ltd., Qingdao, China) was used as control.

#### 4.8.5. Swelling Testing

The swelling ratio of hydrogels was determined using a general gravimetric method [56]. Three sets of disk specimens with a diameter of 1 cm were made from the dry-weighed samples (recorded as m_d_) and then immersed and allowed to soak in PBS (pH 7.4) for 24 h at 37 °C. The samples were removed after the swelling equilibrium was reached, the extra moisture was wiped from the surface of the membrane with filter paper, and, then, they were weighed (recorded as m_s_) again. The tests were performed three times, and the average results were noted. The swelling ratio (%) of the samples was calculated using Formula (5) as the following [57]:

(5) $$Swelling\;ratio\;(\%)=\frac{m_{s}-m_{d}}{m_{d}}\times 100$$

#### 4.8.6. Water Retention Testing

The capacity of water retention was determined according to the following steps [48]: the specimens were submerged in UP water until they reached equilibrium, and the excess water was removed from the surface; the specimen weight was measured and recorded as W_eq_. Then, the wet hydrogel samples were then stored in an incubator at 37 °C. Then, they were weighed at periodic intervals (recorded as W_t_), and the mean values were obtained. The water retention ratio of hydrogel patches was calculated by using Formula (6) as below [23]:

(6) $$Water\;retention\;ratio\;(\%)=\frac{W_{t}}{W_{eq}}\times 100$$

where W_eq_ stands for the initial weight (t = 0 min), and W_t_ is the weight of hydrogels in different times from 1 h to 8 h.

#### 4.8.7. Releasing of Loaded Component from the Hydrogel Patch

According to the previous design for the whole system, the components encapsulated in nanoliposomes can be released from the hydrogel patch and play their skincare role; that is, the hydrogel patch can also act as the carrier for delivering bioactive ingredients. Due to the fact that the two kinds of African essential oils belong to a complex system composed of multiple components, it is not easy to choose a well-recognized single compound for quantitative analysis in the study of releasing behaviors. Therefore, the DES of betaine–phytic acid (1:3) encapsulated together with EOs was chosen as the released object for investigation to demonstrate that the relevant components can be released. Due to being located at a deeper position in liposomes than EOs, it was treated as a marker to monitor the release behavior and overall diffusion the encapsulated components. Based on the developed method using Franz diffusion cell [51], a medical polymethylsiloxane (PDMS) film (BioExcellence^®^ International Tech. Co., Ltd., Beijing, China; thickness: 0.5 mm, area: 1.5 × 1.5 cm^2^) was first soaked in 95% ethanol for 30 min and dried naturally for use. The effective diffusion area was 1.33 cm^2^, and the cell jacket was kept at 32 ± 0.5 °C. The volume of the receiving cell was 15 mL, 0.9% physiological saline (Biosharp Biotechnology Co., Ltd., Hefei, China) was used as the receiving medium, and, then, the permeation experiments were carried out as before. The released amount of DES was detected with the HPLC conditions in Section 4.3. The permeability coefficient was calculated according to previous report [58].

## Figures and Tables

**Table 1:** SPF values of all the samples.

Sample	Λ (nm)	Abs 1	Abs 2	Abs 3	Aver Abs	SD Abs	CF × EE(λ) × I(λ) × Ab (λ)	Total SPF	*p*
AO	290	0.240	0.248	0.248	0.245	0.005	0.037	1.503	<0.01
	295	0.242	0.240	0.243	0.242	0.002	0.197		
	300	0.181	0.179	0.194	0.185	0.008	0.531		
	305	0.141	0.151	0.152	0.148	0.006	0.485		
	310	0.096	0.105	0.094	0.098	0.006	0.183		
	315	0.065	0.075	0.068	0.069	0.005	0.058		
	320	0.057	0.072	0.069	0.066	0.008	0.012		
AO:PFSO = 2:1	290	0.272	0.269	0.264	0.268	0.004	0.040	1.622	<0.01
	295	0.232	0.235	0.236	0.234	0.002	0.191		
	300	0.180	0.190	0.191	0.187	0.006	0.537		
	305	0.167	0.157	0.166	0.163	0.006	0.535		
	310	0.119	0.109	0.113	0.114	0.005	0.212		
	315	0.113	0.101	0.109	0.108	0.006	0.090		
	320	0.081	0.089	0.085	0.085	0.004	0.015		
AO:PFSO = 1:1	290	0.268	0.271	0.278	0.272	0.005	0.041	1.511	<0.01
	295	0.233	0.231	0.234	0.233	0.002	0.190		
	300	0.166	0.175	0.163	0.168	0.006	0.483		
	305	0.135	0.158	0.153	0.149	0.121	0.487		
	310	0.110	0.113	0.107	0.110	0.003	0.205		
	315	0.098	0.109	0.111	0.106	0.007	0.089		
	320	0.084	0.087	0.088	0.086	0.002	0.016		
AO:PFSO = 1:2	290	0.296	0.305	0.299	0.300	0.005	0.045	1.430	<0.01
	295	0.237	0.249	0.237	0.241	0.007	0.197		
	300	0.153	0.148	0.155	0.152	0.004	0.437		
	305	0.132	0.134	0.134	0.133	0.001	0.437		
	310	0.102	0.114	0.111	0.109	0.006	0.203		
	315	0.116	0.106	0.117	0.113	0.006	0.095		
	320	0.096	0.093	0.089	0.093	0.004	0.017		
PFSO	290	0.313	0.308	0.314	0.312	0.003	0.047	1.679	<0.01
	295	0.273	0.274	0.279	0.275	0.003	0.225		
	300	0.172	0.172	0.188	0.177	0.009	0.510		
	305	0.153	0.158	0.159	0.157	0.003	0.514		
	310	0.128	0.137	0.134	0.133	0.005	0.248		
	315	0.126	0.139	0.136	0.134	0.007	0.112		
	320	0.128	0.132	0.134	0.131	0.003	0.024		

**Table 2:** Ingredients of essential oil-DES nanoliposomes.

Ingredients	Physical-Dispersion Method		Alcohol-Injection Method	
	S1	S2	S3	S4
Cholesterol (mg)	100	100	100	100
Lecithin (mg)	100	200	100	200
EOs (g)	1	1	1	1
DES (g)	1	1	1	1
Ethanol (mL)	-	-	7	7
Trichloromethane (mL)	5	5	-	-
Methanol (mL)	-	-	3	3
PBS (mL)	50	50	50	50

## Data Availability

All the data are contained within this article and the Supplementary Materials.

## Supplementary Materials

The following supporting information can be downloaded at https://www.mdpi.com/article/10.3390/gels11050364/s1, Figure S1: FT-IR spectra of betaine (KBr disk); Figure S2: FT-IR spectra of phytic acid (KBr disk); Figure S3: UV spectra of encapsulated system; Table S1: The experimental details for the test on Ca^2+^ combined with oil samples; and Table S2: The values of EE(λ) × I(λ).
