# Supplementary material for: Development of Liposome-Based Hydrogel Patches Incorporating Essential Oils of African Plants and Deep Eutectic Solvents
Source: Gels. 2025 May 15;11(5):364. doi: 10.3390/gels11050364 (PMC12111014; doi:10.3390/gels11050364)
Supplement: Supplementary file 1 [file gels-11-00364-s001.zip › gels-3601629-supplementary.pdf]

Supplemental material

Development of Liposome-Based Hydrogel Patches Incorporating Essential Oils of African Plants and Deep Eutectic Solvents

Wanhang Jiang, Sara Toufouki, Subhan Mahmood, Ali Ahmad, Alula Yohannes, Yang Xiang*, Shun Yao*

School of Chemical Engineering, Sichuan University, Chengdu, 610065, ChinaCollege of Natural Science, Wolkite University, Wolkite City, Ethiopia

Children's Drug Research Institute, Jianmin Group, Wuhan, China

Journal name: Gels

7 pages, 2 tables and 3 figures

*Author for correspondence. Tel.:+86-028-85405221; fax: +86-028-85405221

*E-mail: cusack@scu.edu.cn (S. Yao)

Table of contents

Table S1 **P3**

Table S2 **P4**

Figure S1 **P5**

Figure S2 **P6**

Figure S3 **P7**

Table S1 The experimental details for the test on Ca^{2+} combined with oil samples

Samples	AO	2:1	1:1	1:2	PFSO
Used EDTA volume (L)	1.10×10^{-3}	0.95×10^{-3}	0.80×10^{-3}	1.15×10^{-3}	1.20×10^{-3}
Used EDTA moles (mol)	1.10×10^{-5}	9.50×10^{-6}	8.00×10^{-6}	1.15×10^{-5}	1.24×10^{-5}
Used Ca^{2+} moles (mol)	2.20×10^{-3}	1.90×10^{-3}	1.60×10^{-3}	2.30×10^{-3}	2.40×10^{-3}
Residual Ca^{2+} mass (mg)	88.17	76.15	64.13	92.18	96.20

Table S2 The values of EE(λ) × I(λ)

λ (nm)	EE(λ) × I(λ)
290	0.0150
295	0.0817
300	0.2874
305	0.3278
310	0.1864
315	0.0839
320	0.0180

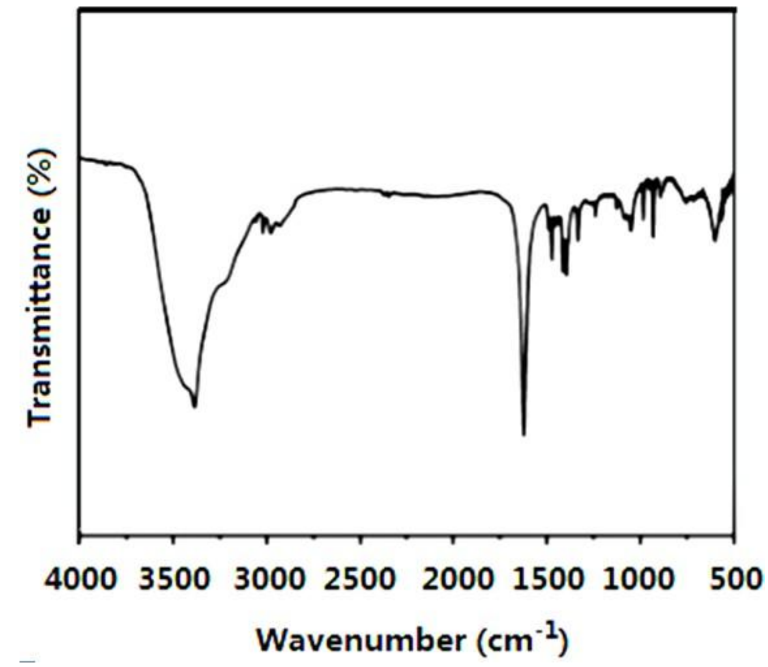

Figure S1 FT-IR spectra of betaine (KBr disc)

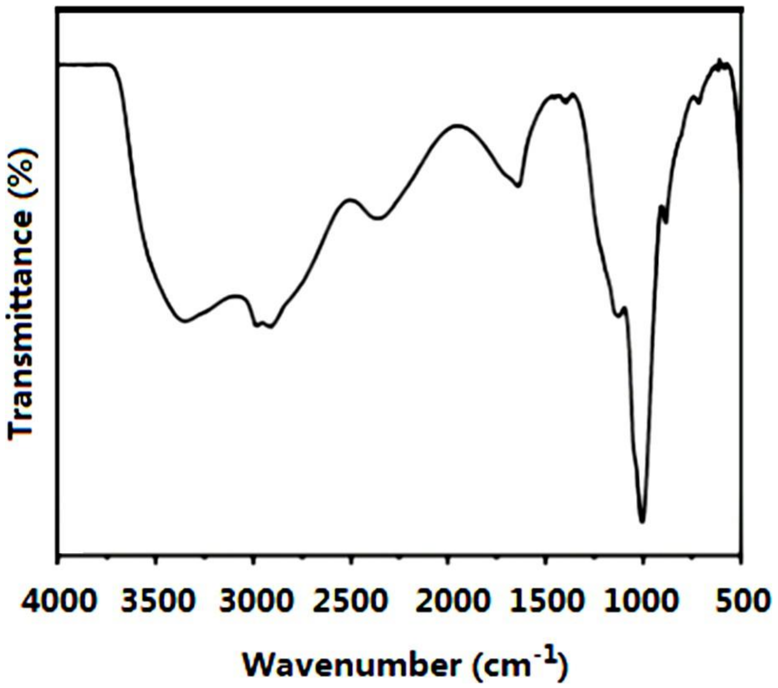

Figure S2 FT-IR spectra of phytic acid (KBr disc)

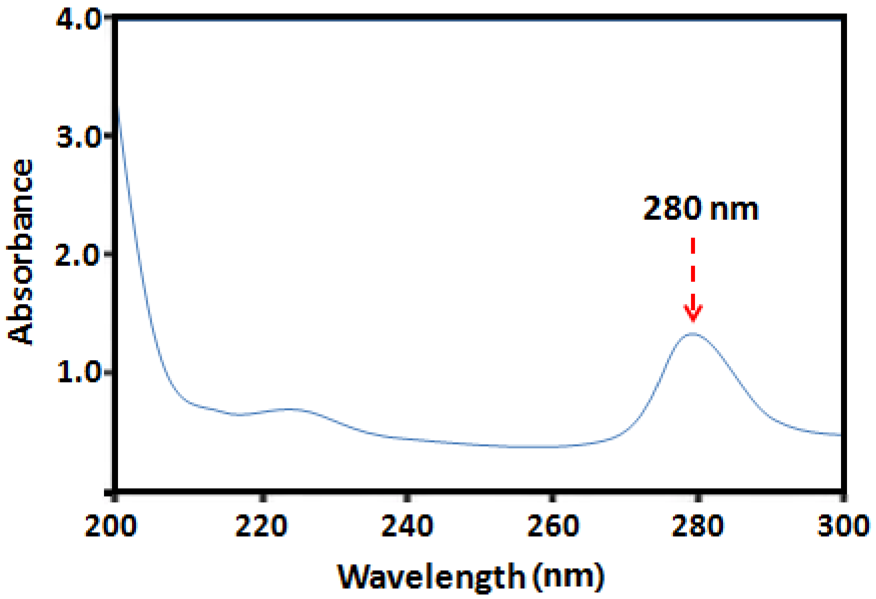

Figure S3 UV spectra of encapsulated system
